# Imatinib and polypharmacy in very old patients with chronic myeloid leukemia: effects on response rate, toxicity and outcome

**DOI:** 10.18632/oncotarget.11657

**Published:** 2016-08-27

**Authors:** Alessandra Iurlo, Alessandro Nobili, Roberto Latagliata, Cristina Bucelli, Fausto Castagnetti, Massimo Breccia, Elisabetta Abruzzese, Daniele Cattaneo, Carmen Fava, Dario Ferrero, Antonella Gozzini, Massimiliano Bonifacio, Mario Tiribelli, Patrizia Pregno, Fabio Stagno, Paolo Vigneri, Mario Annunziata, Francesco Cavazzini, Gianni Binotto, Giovanna Mansueto, Sabina Russo, Franca Falzetti, Enrico Montefusco, Gabriele Gugliotta, Sergio Storti, Ada M. D'Addosio, Luigi Scaffidi, Laura Cortesi, Michele Cedrone, Antonella Russo Rossi, Paolo Avanzini, Endri Mauro, Antonio Spadea, Francesca Celesti, Gianfranco Giglio, Alessandro Isidori, Monica Crugnola, Elisabetta Calistri, Federica Sorà, Giovanna Rege-Cambrin, Simona Sica, Luigiana Luciano, Sara Galimberti, Ester M. Orlandi, Monica Bocchia, Mauro Tettamanti, Giuliana Alimena, Giuseppe Saglio, Gianantonio Rosti, Pier Mannuccio Mannucci, Agostino Cortelezzi

**Affiliations:** ^1^ Oncohematology Division, IRCCS Ca' Granda - Maggiore Policlinico Hospital Foundation, University of Milan, Milan, Italy; ^2^ Department of Neuroscience, IRCCS - Istituto di Ricerche Farmacologiche “Mario Negri”, Milan, Italy; ^3^ Department of Cellular Biotechnologies and Hematology, University “La Sapienza” of Rome, Rome, Italy; ^4^ Institute of Hematology “L. and A. Seràgnoli”, Department of Experimental, Diagnostic and Specialty Medicine, “S. Orsola-Malpighi” University Hospital, University of Bologna, Bologna, Italy; ^5^ Hematology Unit, Sant'Eugenio Hospital, Rome, Italy; ^6^ Division of Hematology and Internal Medicine, University of Turin, “San Luigi Gonzaga” University Hospital, Orbassano, Turin, Italy; ^7^ Hematology Unit, University of Turin, Turin, Italy; ^8^ Haematology, AOU Careggi, University of Firenze, Firenze, Italy; ^9^ Department of Medicine, Section of Hematology, University of Verona, Verona, Italy; ^10^ Division of Hematology and BMT, Azienda Ospedaliero - Universitaria di Udine, Udine, Italy; ^11^ Hematology Unit, Azienda Ospedaliero Universitaria Città della Salute e della Scienza, Turin, Italy; ^12^ Hematology Unit, Ferrarotto Hospital, Catania, Italy; ^13^ Hematology Unit, Cardarelli Hospital, Naples, Italy; ^14^ Hematology Unit, University of Ferrara, Ferrara, Italy; ^15^ Hematology Unit, University of Padova, Padova, Italy; ^16^ Department of Onco-Hematology, IRCCS-CROB, Rionero in Vulture, Italy; ^17^ Hematology Unit, AOU G. Martino, Messina, Italy; ^18^ Division of Hematology and Clinical Immunology, Department of Medicine, University of Perugia, Perugia, Italy; ^19^ Hematology Unit, Sant'Andrea Hospital, Rome, Italy; ^20^ Onco-Hematology Unit, Università Cattolica Giovanni Paolo II, Campobasso, Italy; ^21^ Immunohematology and Trasfusional Medicine Division, S. Pietro Fatebenefratelli Hospital, Rome, Italy; ^22^ Hematology Unit, S. Giovanni Hospital, Rome, Italy; ^23^ Hematology and Transplants Unit, University of Bari, Bari, Italy; ^24^ Hematology, Arcispedale Santa Maria Nuova - IRCCS, Reggio Emilia, Italy; ^25^ Department of Internal Medicine, Pordenone General Hospital, Pordenone, Italy; ^26^ Hematology and Stem Cell Transplantation Unit, Regina Elena Institute, Rome, Italy; ^27^ Hematology Unit, Belcolle Hospital, Viterbo, Italy; ^28^ Hematology Unit, Campobasso Hospital, Campobasso, Italy; ^29^ Hematology Unit, Pesaro Hospital, Pesaro, Italy; ^30^ Hematology and BMT Unit, Azienda Ospedaliero-Universitaria di Parma, Parma, Italy; ^31^ Hematology Unit, Treviso Hospital, Treviso, Italy; ^32^ Institute of Hematology, Università Cattolica Sacro Cuore, Rome, Italy; ^33^ Hematology Unit, “Federico II” Hospital, University of Naples, Naples, Italy; ^34^ Department of Clinical and Experimental Medicine, Section of Hematology - University of Pisa, Pisa, Italy; ^35^ Oncology-Hematology Department, Hematology Unit, Fondazione IRCCS Policlinico San Matteo, Pavia, Italy; ^36^ Hematology Unit, Azienda Ospedaliera Universitaria Senese and University of Siena, Siena, Italy; ^37^ Angelo Bianchi Bonomi Hemophilia and Thrombosis Center, IRCCS Ca' Granda - Maggiore Policlinico Hospital Foundation and University of Milan, Milan, Italy

**Keywords:** chronic myeloid leukemia, comorbidities, imatinib, old patients, polypharmacy

## Abstract

**Background:**

About 40% of all patients with chronic myeloid leukemia are currently old or very old. They are effectively treated with imatinib, even though underrepresented in clinical studies. Furthermore, as it happens in the general population, they often receive multiple drugs for associated chronic illnesses. Aim of this study was to assess whether or not in imatinib-treated patients aged >75 years the exposure to polypharmacy (5 drugs or more) had an impact on cytogenetic and molecular response rates, event-free and overall survival, as well as on hematological or extra-hematological toxicity.

**Methods:**

296 patients at 35 Italian hematological institutions were evaluated.

**Results:**

Polypharmacy was reported in 107 patients (36.1%), and drugs more frequently used were antiplatelets, diuretics, proton pump inhibitors, ACE-inhibitors, beta-blockers, calcium channel blockers, angiotensin II receptors blockers, statins, oral hypoglycemic drugs and alpha blockers. Complete cytogenetic response was obtained in 174 patients (58.8%), 78 (26.4%) within 6 month, 63 (21.3%) between 7 and 12 months. Major molecular response was obtained in 153 patients (51.7%), 64 (21.6%) within the 12 month. One hundred and twenty-eight cases (43.2%) of hematological toxicity were recorded, together with 167 cases (56.4%) of extra-hematological toxicity. Comparing patients exposed to polypharmacy to those without, no difference was observed pertaining to the dosage of imatinib, cytogenetic and molecular responses and hematological and extra-hematological toxicity.

**Conclusion:**

Notwithstanding the several interactions reported in the literature between imatinib and some of the medications considered herewith, this fact does not seem to have a clinical impact on response rate and outcome.

## INTRODUCTION

Chronic myeloid leukemia (CML) has an annual incidence of 1.1-1.8 cases per 100,000 adults, accounting for 15-20% of newly diagnosed cases of leukemia in adults [1]. The median age at diagnosis is 50-60 years, with a peak at 70-74 years [2]. Thus, a significant proportion of CML patients are old or very old. In the past the primary aim of treatment in this subset of patients was to contain the leukemic mass, because older patients had no access to appropriate therapies like interferon or allogeneic bone marrow transplantation [3]. Patients' prognosis changed dramatically with the development of tyrosine kinase inhibitors (TKIs). This targeted approach has markedly increased the survival of CML patients, which is now approaching that of the general population [1, 4, 5].

Even though a relevant proportion of CML patients are old or very old, they are generally under-represented in clinical trials [2]. Moreover, older TKI-treated patients suffer from comorbidities, whose number is usually higher in patients aged 75 years or more [6]. Several studies have established that multimorbidity is an independent predictor of all-cause mortality [7–9]: more specifically, a recent study by Saussele et al. [6] on the influence of comorbidities on the outcome of 1519 CML patients found a strong negative association between comorbidities at diagnosis and overall survival (OS), even though comorbidities had no impact on treatment success. Furthermore, Efficace et al. [10] reported that comorbidities have a critical impact also on the health-related quality of life of these patients, particularly on general health, pain, physical functioning and vitality.

Due to multiple concomitant diseases, medication intake usually increases in older people, so that polypharmacy, a well-known risk factor for drug-drug interactions (DDIs) and adverse drug reactions (ADR), is highly prevalent. In older people, these risks are also increased because ageing is often accompanied by physiological changes in the pharmacokinetic (PK) and pharmacodynamic processes [11, 12]. In older CML cases exposed to polypharmacy, the use of TKIs may be another risk factor for DDIs, because these drugs are extensively metabolized by cytochrome (CYP) P450.

In addition, some TKIs are substrates or inhibitors of the drug transporter P-glycoprotein (PgP) and the organic cation transporter 1 (hOCT1). In particular, imatinib is metabolized primarily by the CYP 3A4 isoenzyme and is a substrate for hOCT1, PgP and breast cancer resistance protein (BCRP). The classes of drugs most commonly involved in DDIs with TKIs, and specifically with imatinib, are anticoagulants, proton pump inhibitors (PPI), antiplatelet agents, calcium channel blockers, statins, antiarrhythmic drugs, beta-blockers, digoxin, macrolides, azoles, rifampicin, dexamethasone and levothyroxine [13]. As a result of the effect of potential DDIs between concomitantly administered drugs, a standard regimen of TKIs may produce different levels of circulating and intra-cellular drug concentrations. Thus, a patient could on one hand have a low response to TKI due to subtherapeutic drug exposure or on the other hand develop TKI toxicity in case of overexposure. However, data specifically on concomitant medications in CML are not reported in the literature.

With this background, the aim of this study was to assess if in CML patients aged 75 years or more and imatinib-treated the exposure to polypharmacy had an impact on such main clinical outcomes as cytogenetic and molecular response rates, event-free survival (EFS) and OS, as well as on hematological or extra-hematological toxicity.

## RESULTS

Two hundred and ninety-six very old patient with chronic-phase CML treated with imatinib were evaluated. Table 1 summarizes the characteristics of the whole cohort and of patients exposed to polypharmacy (5 or more drugs) compared with those not exposed to polypharmacy (less than 5 drugs).

In the overall cohort, mean age at diagnosis was 79.4 (± 3.7 SD) years, 152 (51.4%) were males, 83 (28%) were Sokal high risk and 103 (34.8%) were treated with reduced dose imatinib (< 400 mg/day). Seventy-one patients (24%) received other treatments before imatinib, mainly hydroxyurea (62 patients; 20.9%) and/or interferon (9 patients; 3%).

Polypharmacy was present in 107 patients (36.1%), and the concomitant drugs more frequently used were antiplatelet agents taken by 152 patients (51.4%), followed by diuretics and PPIs in 125 (42.3%), ACE-inhibitors in 83 (28%), beta-blockers in 61 (20.6%), calcium channel blockers in 59 (19.9%), angiotensin II receptor blockers (ARB) in 57 (19.3%), statins in 44 (14.9%), oral hypoglycemic drugs in 38 (12.8%) and alpha blockers in 31 (10.5%).

Complete cytogenetic response was obtained in 174 patients (58.8%), 78 (26.4%) within 6 month, 63 (21.3%) between 7 and 12 months, and the remaining patients after 12 months. Major molecular response (MMR) was obtained in 153 patients (51.7%), of whom 64 (21.6%) within the 12^th^ month. However, differently from molecular analyses, a cytogenetic evaluation was not available in 58 patients (19.6%). One hundred and twenty-eight cases (43.2%) of hematological toxicity were recorded, together with 167 cases (56.4%) of extra-hematological toxicity.

Comparing patients exposed to polypharmacy with those without polypharmacy, a statistically significant difference between the two groups was observed for age and comorbidities. No difference was found in the two groups pertaining to the dosage of imatinib, cytogenetic and molecular responses and hematological and extra-hematological toxicity (Table 1).

**Table 1:** General characteristics of 296 chronic-phase CML patients, overall and according to the exposure or not to polypharmacy

Variables	Overall cohort (n. 296)	%	No polypharmacy (A) (N of drugs 0-4) (n. 189)	%	On polypharmacy (B) (N of drugs >5) (n. 107)	%	A*vs* B *p* value
Male, n (%)	152	51.4	95	50.3	57	53.3	0.61
Mean age ± SD	79.4 ± 3.7		79.0 ± 3.6		80.1 ± 3.7		0.01
Clinical parameters							
Sokal score, n (%)							
Low	4	1.4	3	1.6	1	0.9	0.83
Intermediate	187	63.2	116	61.4	71	66.4	
High	83	28.0	55	29.1	28	26.2	
Not available	22	7.4	15	7.9	7	6.5	
Imatinib dosage, n (%)							
<400 mg/day	103	34.8	61	32.3	42	39.3	0.22
>400 mg/day	193	65.2	128	67.7	65	60.7	
CCI, n (%)							
0	105	35.5	79	41.8	26	24.3	0.004
1	74	25.0	47	24.9	27	25.2	
2+	117	39.5	63	33.3	54	50.5	
Concomitant drugs							
Antihypertensives, n (%)	214	72.3	117	61.9	97	90.7	<0.0001
Diuretics, n (%)	123	41.6	51	27.0	72	67.3	<0.0001
ACE inhibitors, n (%)	81	27.4	40	21.2	41	38.3	0.0015
Beta-blockers, n (%)	59	19.9	18	9.5	41	38.3	<0.0001
Calcium channel blockers, n (%)	57	19.3	25	13.2	32	29.9	0.0005
Angiotensin II receptor blockers, n (%)	55	18.6	24	12.7	31	29.0	0.0005
Alpha blockers, n (%)	27	9.1	9	4.8	18	16.8	0.0005
Antiplatelet agents, n (%)	144	48.6	70	37.0	74	69.2	<0.0001
Proton pump inhibitors, n (%)	122	41.2	52	27.5	70	65.4	<0.0001
Statins, n (%)	43	14.5	15	7.9	28	26.2	<0.0001
Oral hypoglycemic drugs, n (%)	40	13.5	18	9.5	22	20.6	0.007
Clinical outcomes							
CCyR, n (%)	174	58.8	113	59.8	61	57.0	0.69
CCyR within 6 months, n (%)	78	26.4	49	25.9	29	27.1	0.57
CCyR 7 to 12 months, n (%)	63	21.3	42	22.2	21	19.6	0.80
MMR, n (%)	153	51.7	93	49.2	60	56.1	0.49
Hematological toxicity, n (%)	126	42.6	79	41.8	47	43.9	0.72
Extra-hematological toxicity, n (%)	167	56.4	107	56.6	60	56.1	0.92

Abbreviations: SD=standard deviation; CCI=Charlson Comorbidity Index; CCyR=Complete Cytogenetic Response; MMR=Major Molecular Response.

According to the starting dosage of imatinib, 193 patients (65.2%) were treated with 400 mg/day or more. The comparison of characteristics of patients treated with less than 400 mg/day and those treated with 400 mg/day or more showed a statistically significant difference only for age, concomitant use of antiplatelet and oral hypoglycemic agents, as well as for cytogenetic response by 12 months (Table 1S).

**Figure 1:**
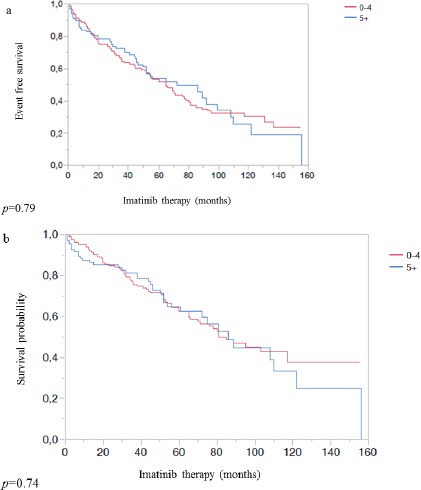
**a.** Event-free survival according to the exposure or not to polypharmacy. **b**. Overall survival according to the exposure or not to polypharmacy.

At multivariable logistic regression analysis (Table 2), polypharmacy was not associated at any time point with cytogenetic response, molecular response and hematological and extra-hematological toxicity. Furthermore, the exposure to polypharmacy failed to affect patient outcomes (EFS *p* = 0.79; OS *p* = 0.74) (Figure 1a and 1b), even though we confirmed the impact that comorbidities had on EFS *(p* = 0.001) or OS (*p* < 0.001) (Figure 1S.a and 1S.b).

Finally, the exposure to multiple concomitant drugs (in particular, classes of antihypertensives, antiplatelet agents, PPIs, statins or oral hypoglycemic drugs) had no statistically significant effect on OS, EFS, cytogenetic response at any time-point, molecular response and hematological and extra-hematological toxicity (Table 2S).

**Table 2:** Response rate and toxicity of 296 chronic-phase CML patients aged 75 years or older according to the exposure or not to polypharmacy

Clinical outcomes	Patients (n. 296)			
	No polypharmacy (N of drugs 0-4) (n. 192)	Polypharmacy (N of drugs >5) (n. 104)	Univariate OR (95% CI)	Multivariable* OR (95% CI)
CCyR within 6 months, n (%)	50 (26)	28 (26.9)	0.81 (0.46-1.43)	0.96 (0.86-1.09)
CCyR 7 to 12 months, n (%)	43 (22.4)	20 (19.2)	0.91 (0.53-1.56)	0.96 (0.86-1.08)
MMR, n (%)	93 (48.4)	60 (57.7)	0.84 (0.51-1.38)	1.00 (0.90-1.11)
Hematological toxicity, n (%)	80 (41.7)	46 (44.2)	0.93 (0.57-1.52)	1.03 (0.93-1.14)
Extra-hematological toxicity, n (%)	108 (56.3)	59 (56.7)	1.33 (0.82-2.17)	1.05 (0.95-1.17)

Abbreviations: OR=Odds Ratio; CCyR=complete cytogenetic response; MMR=major molecular response.

* Adjusted for age and sex.

## DISCUSSION

Imatinib is a very effective treatment also for older CML patients in chronic phase, allowing high response rates and survival, comparable to those observed in the younger [14]. However, several older patients actually receive a starting dose of imatinib < 400 mg/day, i.e. lower than the standard dose, owing to worse performance status compared to younger ones [15].

While the prognostic impact of comorbidities on CML has been previously studied, establishing their strong negative influence [6, 16], there are no data concerning the role that multiple drugs may have regarding response rate or outcome during imatinib therapy in very old CML patients. Indeed, there is a list of observed or potential DDIs between first and second generation TKIs (imatinib, dasatinib and nilotinib) and commonly used drugs which are concomitantly prescribed [13, 17], but this list is mainly the result of meta-analyses regarding *in vitro* studies rather than of *in vivo* studies. To address this issue, we retrospectively evaluated a large cohort of 296 patients from 35 Italian hematological centers aged 75 years or more at the time of imatinib start. Our data show that in 36.1% of these cases 5 or more concomitant drugs were regularly co-prescribed, and that diuretics, beta-blockers and statins were the most frequently used [18].

The achievement of CCyR at 6 and 12 months and of MMR were not influenced by the number and type of concomitant drugs. Similarly, the incidence and type of reported ADRs were not apparently influenced by the type and number of co-treatments. This is perhaps due to the variety of conditions in which combination therapies were analyzed, adding a level of treatment complexity owing to overlapping PK interactions (i.e. absorption, distribution, metabolism) [19]. Most importantly, at variance with comorbidities that did significantly influence patient outcome also in the present cohort, concomitant drugs and single classes of drugs failed to affect EFS or OS in old CML patients.

This is the first study which specifically considers the prognostic significance of TKIs and concomitant drug exposure in a large population of very old CML patients outside the context of a prospective clinical trial. However, we are aware that our study has some limitations: namely, its retrospective nature and the lack of information regarding the severity of the comorbidities, the latter due to the fact that they were evaluated by means of Charlson Comorbidity Index (CCI).

Notwithstanding the several PK interactions reported in the literature between imatinib and some of the medications considered herewith [13, 17], this does not seem to have a clinical impact on the response rate and outcome also in a real-life setting. Thus, we believe that an older age and a worse performance status are still fully compatible with TKIs use (and effectiveness), at variance with more aggressive cytotoxic treatments actually used in other hematological malignancies. Our findings may represent useful information for clinicians, allowing them to manage their very old CML patients in chronic phase in the best possible way. More specifically, it should be further considered that efficacy and safety of different co-prescribed drugs can be up- or down-regulated by imatinib: for example, imatinib enhances the pharmacologic effects and possibly toxicity of cyclosporin, simvastatin, metoprolol, calcium channel blockers such as verapamil and diltiazem, amiodarone, and quinidine [13]. Therefore, in patients taking imatinib, these drugs should be either tapered or replaced by safer alternatives.

In conclusion, the prognosis of very old CML cases depended more on their comorbidities than on the number of drugs taken. Furthermore, a larger number of CML patients will have a life expectancy similar to that of general population, and this mainly due to TKIs treatment.

With this study we did underline the safety and efficacy of imatinib also in this subset of patients. It could be interesting to evaluate also the impact of second generation TKIs (dasatinib, nilotinib and bosutinib).

## MATERIALS AND METHODS

### Patients

In the frame of this observational study 35 Italian hematological centers were asked to retrospectively collect data on all CML patients on chronic phase admitted from January 2002 to October 2015, provided they were aged 75 years or more at the time of imatinib start.

### Methods

All the following data were collected at baseline before imatinib initiation: sociodemographic and hematological variables, type and number of concomitant diseases and drugs and initial dose of imatinib. Any diagnosed clinical condition requiring a specific and chronic treatment was considered a concomitant disease and assessed as defined in the CCI [20]. Hematological and extra-hematological toxicities were graded according to the WHO scale, but for the purpose of this study only severe toxicities were considered in the analysis.

We defined polypharmacy according to the REPOSI registry as exposure of a CML patient to five or more different medications in addition to imatinib [21]. In particular, we analyzed the effects of drugs that may have a potential risk of interaction with imatinib, such as antihypertensives, ACE-inhibitors, beta-blockers, calcium channel blockers, ARB, antiplatelet agents, PPI, statins and oral hypoglycemic drugs [13].

Monitoring and responses followed the current European LeukemiaNet recommendations [22–24].

Overall survival was calculated from the date of imatinib start until death due to any cause. Event-free survival was calculated from the date of imatinib start to any of the following events: primary hematological resistance to imatinib, definitive imatinib discontinuation due to toxicity or any other cause, secondary hematological or cytogenetic resistance to imatinib or death due to any cause.

### Statistical analysis

Clinical and sociodemographic characteristics of the sample were described using absolute numbers and percentages, except that for age mean and standard deviation (SD) were used. Comparison between subgroups with and without polypharmacy, or with dosage of imatinib lower/higher than 400 mg/day were performed by means of chi square test for all variables except for age, where a *t*-test was used. The univariable association between polypharmacy and CCyR (0 to 6 months, 7 to 12 months, more than 12 months) was studied using an ordinal logistic regression model and reporting odds ratios (ORs) and 95% confidence intervals (CIs). The associations between polypharmacy and molecular response (MR), hematological or extra-hematological toxicity were studied using nominal logistic regression models and reporting ORs and 95% CIs. Multivariable ordinal/nominal logistic regressions for all models were performed correcting for age and sex. Kaplan-Meier curves were plotted to analyze differences in survival from the beginning of imatinib therapy to the occurrence of OS or EFS by presence of polypharmacy, testing differences by means of log-rank tests. Cox proportional hazards models were used to obtain the hazard ratios (HRs) of OS or EFS for polypharmacy in a multivariable models (adjusting for age and sex). Single drug class associations with CCyR, MR, toxicity, OS or EFS were analyzed as reported above for polypharmacy. Categorized age was used in multivariable models to correct for imbalances. Statistical analyses were performed using JMP Pro 12.1 (SAS Institute Inc.).

## SUPPLEMENTARY MATERIAL


