# Supplementary material for: Imatinib and polypharmacy in very old patients with chronic myeloid leukemia: effects on response rate, toxicity and outcome
Source: Oncotarget. 2016 Aug 27;7(48):80083–90. doi: 10.18632/oncotarget.11657 (PMC5346773; doi:10.18632/oncotarget.11657)
Supplement: Supplementary file 1 [file oncotarget-07-80083-s001.pdf]

Imatinib and polypharmacy in very old patients with chronic myeloid leukemia: effects on response rate, toxicity and outcome

Supplementary Material

Table 1S. General characteristics of 296 chronic-phase CML patients according to the imatinib dosage.

Variables	Overall cohort (n. 296)	%	Dosage <400 mg/day (A) (n. 103)	%	Dosage ≥400 mg/day (B) (n. 193)	%	A *vs* B *p* value
**Male, n (%)**	152	51.4	48	46.6	104	53.9	0.23
**Mean age ± SD**	79.4 ± 3.7		81.1 ± 3.9		78.5 ± 3.2		<0.0001
**Clinical parameters**							
Sokal score, n (%)							
Low	4	1.4	1	1.0	3	1.6	0.09
Intermediate	187	63.2	56	54.4	131	67.9	
High	83	28.0	35	34.0	48	24.9	
Not available	22	7.4	11	10.7	11	5.7	
CCI, n (%)							
0	105	35.5	28	27.2	77	39.9	0.07
1	74	25.0	31	30.1	43	22.3	
2+	117	39.5	44	42.7	73	37.8	
Polypharmacy, n (%)							
0-4	189	63.9	61	59.2	128	66.3	0.2
≥5	107	36.1	42	40.8	65	33.7	
**Concomitant drugs**							
Antihypertensives, n (%)	214	72.3	73	70.9	141	73.1	0.68
Diuretics, n (%)	123	41.6	40	38.8	83	43.0	0.48
ACE inhibitors, n (%)	81	27.4	33	32.0	48	24.9	0.18
Beta-blockers, n (%)	59	19.9	23	22.3	36	18.7	0.45
Calcium channel blockers, n (%)	57	19.3	23	22.3	34	17.6	0.32
Angiotensin II receptor blockers, n (%)	55	18.6	16	15.5	39	20.2	0.32
Alpha blockers, n (%)	27	9.1	9	8.7	18	9.3	0.86
Antiplatelet agents, n (%)	144	48.6	61	59.2	83	43.0	0.007
Proton pump inhibitors, n (%)	122	41.2	46	44.7	76	39.4	0.37
Statins, n (%)	43	14.5	18	17.5	25	13.0	0.29
Oral hypoglycemic drugs, n (%)	40	13.5	22	21.4	18	9.3	0.003
**Clinical outcomes**							
CCyR, n (%)	174	58.8	50	48.5	124	64.2	0.006
CCyR within 6 months, n (%)	78	26.4	16	15.5	62	32.1	0.015
CCyR 7 to 12 months, n (%)	63	21.3	24	23.3	39	20.2	0.04
MMR, n (%)	153	51.7	45	43.7	108	56.0	0.21
Hematological toxicity, n (%)	126	42.6	41	39.8	85	44.0	0.48

Extra-hematological toxicity, n (%)	167	56.4	55	53.4	112	58.0	0.44

Abbreviations: SD=standard deviation; CCI=Charlson Comorbidity Index; CCyR=Complete Cytogenetic Response; MMR=Major Molecular Response.

Table 2S. Analysis of the effects of exposure to concomitant drugs on main clinical outcomes. All analysis adjusted for sex and age.

Table 2S. a: Overall survival and event-free survival.

	OVERALL SURVIVAL		EVENT-FREE SURVIVAL	
	HR (CI 95%)	*p value*	HR (CI 95%)	*p value*
**Antihypertensive drugs**	0.95 (0.63-1.46)	0.82	0.91 (0.64-1.29)	0.58
**Diuretics**	0.96 (0.65-1.42)	0.86	0.86 (0.61-1.19)	0.36
**ACE inhibitors**	1.00 (0.64-1.52)	1.00	0.95 (0.65-1.35)	0.78
**Beta-blockers**	1.33 (0.81-2.09)	0.25	1.09 (0.71-1.63)	0.68
**Calcium channel blockers**	1.08 (0.65-1.72)	0.75	1.02 (0.66-1.53)	0.92
**Angiotensin II receptor blockers**	0.63 (0.35-1.06)	0.08	0.71 (0.44-1.08)	0.11
**Alpha blockers**	0.71 (0.27-1.50)	0.40	0.93 (0.47-1.66)	0.82
**Antiplatelet agents**	0.96 (0.65-1.41)	0.83	0.85 (0.61-1.17)	0.31
**Proton pump inhibitors**	1.05 (0.71-1.53)	0.82	1.12 (0.81-1.54)	0.49
**Statins**	0.82 (0.40-1.51)	0.55	0.97 (0.56-1.57)	0.91
**Oral hypoglycemic drugs**	1.18 (0.63-2.03)	0.59	1.28 (0.77-2.00)	0.33

Table 2S. b: CCyR within 6 months and 7 to 12 months.

	CCyR within 6 months		CCyR 7 to 12 months	
	OR (CI 95%)	*p value*	OR (CI 95%)	*p value*
**Antihypertensive drugs**	1.17 (0.82-1.67)	0.38	1.12 (0.77-1.63)	0.56
**Diuretics**	1.01 (0.75-1.38)	0.94	1.11 (0.80-1.55)	0.54
**ACE inhibitors**	1.02 (0.73-1.44)	0.89	1.22 (0.84-1.80)	0.31
**Beta-blockers**	0.90 (0.62-1.32)	0.58	1.04 (0.70-1.58)	0.84
**Calcium channel blockers**	1.22 (0.82-1.86)	0.34	0.95 (0.64-1.43)	0.80
**Angiotensin II receptor blockers**	0.70 (0.47-1.04)	0.08	0.86 (0.56-1.32)	0.50
**Alpha blockers**	0.91 (0.56-1.49)	0.70	1.71 (0.85-4.42)	0.18
**Antiplatelet agents**	0.75 (0.55-1.02)	0.07	0.66 (0.47-0.92)	0.01
**Proton pump inhibitors**	0.85 (0.62-1.17)	0.32	0.78 (0.56-1.08)	0.14
**Statins**	0.77 (0.50-1.19)	0.25	0.90 (0.55-1.50)	0.68
**Oral hypoglycemic drugs**	1.11 (0.68-1.88)	0.68	0.77 (0.49-1.21)	0.25

Table 2S. c: Major molecular response (MMR) at any time point.

	MMR at any time-point	
	OR (CI 95%)	*p value*
**Antihypertensives**	1.06 (0.62-1.85)	0.83
**Diuretics**	0.86 (0.52-1.40)	0.55
**ACE inhibitors**	0.80 (0.46-1.38)	0.43
**Beta-blockers**	0.96 (0.50-1.77)	0.89
**Calcium channel blockers**	1.51 (0.83-2.74)	0.18
**Angiotensin II receptor blockers**	0.53 (0.26-1.03)	0.06
**Alpha blockers**	1.41 (0.61-3.16)	0.41
**Antiplatelet agents**	1.06 (0.65-1.72)	0.83
**Proton pump inhibitors**	0.84 (0.51-1.37)	0.48
**Statins**	1.07 (0.54-2.08)	0.84
**Oral hypoglycemic drugs**	1.15 (0.56-2.28)	0.70

Table 2S. d: Hematological toxicity and extra-hematological toxicity.

	Hematological toxicity		Extra-hematological toxicity	
	OR (CI 95%)	*p value*	OR (CI 95%)	*p value*
**Antihypertensives**	0.88 (0.51-1.51)	0.65	0.95 (0.56-1.61)	0.86
**Diuretics**	1.07 (0.65-1.75)	0.79	0.86 (0.53-1.38)	0.52
**ACE inhibitors**	0.89 (0.52-1.52)	0.66	1.30 (0.77-2.22)	0.33
**Beta-blockers**	1.50 (0.81-2.86)	0.20	0.80 (0.44-1.44)	0.45
**Calcium channel blockers**	1.03 (0.56-1.93)	0.93	0.99 (0.55-1.80)	0.97
**Angiotensin II receptor blockers**	1.41 (0.75-2.69)	0.29	1.00 (0.55-1.84)	0.99
**Alpha blockers**	1.13 (0.49-2.79)	0.78	1.24 (0.55-2.93)	0.61
**Antiplatelet agents**	1.15 (0.70-1.87)	0.58	0.94 (0.59-1.51)	0.81
**Proton pump inhibitors**	1.30 (0.79-2.14)	0.30	1.30 (0.81-2.09)	0.29
**Statins**	0.98 (0.50-1.96)	0.95	1.14 (0.59-2.26)	0.69
**Oral hypoglycemic drugs**	1.23 (0.61-2.54)	0.56	1.02 (0.52-2.04)	0.95

Abbreviations: CCyR=Complete Cytogenetic Response; MMR=Major Molecular Response.

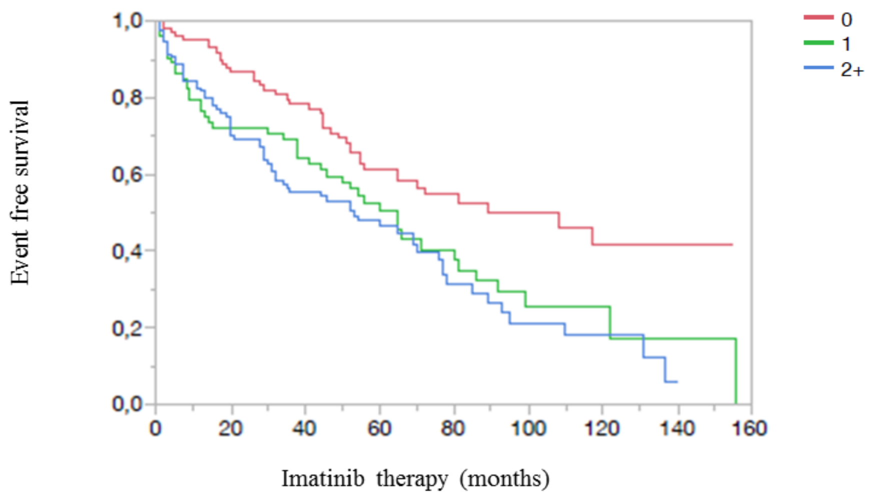

p=0.001

Figure 1S.a. Event-free survival according to the Charlson Comorbidity Index.

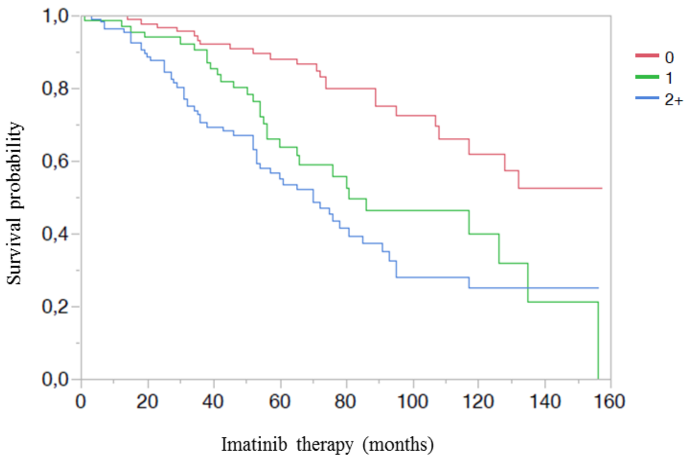

p<0.001

Figure 1S.b. Overall survival according to the Charlson Comorbidity Index.
